# Optimization of singlet oxygen production from photosensitizer‐incorporated, medically relevant hydrogels

**DOI:** 10.1002/jbm.b.33562

**Published:** 2015-10-27

**Authors:** Áine T. De Baróid, Colin P. McCoy, Rebecca A. Craig, Louise Carson, Gavin P. Andrews, David S. Jones, Sean P. Gorman

**Affiliations:** ^1^ School of Pharmacy, Queen's University BelfastBelfastBT9 7BLUK

**Keywords:** fluence, ADPA, PACT, singlet oxygen

## Abstract

Photodynamic therapy and photodynamic antimicrobial chemotherapy are widely used, but despite this, the relationships between fluence, wavelength of irradiation and singlet oxygen (^1^O_2_) production are poorly understood. To establish the relationships between these factors in medically relevant materials, the effect of fluence on ^1^O_2_ production from a tetrakis(4‐*N*‐methylpyridyl)porphyrin (TMPyP)‐incorporated 2‐hydroxyethyl methacrylate: methyl methacrylate: methacrylic acid (HEMA: MMA:MAA) copolymer, a total energy of 50.48 J/cm^2^, was applied at varying illumination power, and times. ^1^O_2_ production was characterized using anthracene‐9,10‐dipropionic acid, disodium salt (ADPA) using a recently described method. Using two light sources, a white LED array and a white halogen source, the LED array was found to produce less ^1^O_2_ than the halogen source when the same power (over 500 − 600 nm) and time conditions were applied. Importantly, it showed that the longest wavelength Q band (590 nm) is primarily responsible for ^1^O_2_ generation, and that a linear relationship exists between increasing power and time and the production of singlet oxygen. © 2015 Wiley Periodicals, Inc. J Biomed Mater Res Part B: Appl Biomater, 105B: 320–326, 2017.

## INTRODUCTION

Photodynamic antimicrobial chemotherapy (PACT) exploits the production of singlet oxygen (^1^O_2_) that occurs when a photosensitizer (PS) is illuminated in the presence of oxygen, to either prevent or reduce the adherence of bacteria to a surface. The ground state PS (*S*_0_) absorbs light energy and is excited into the singlet state (*S*_1_). This is then transformed, by intersystem crossing, to the excited triplet state, which reacts with molecular oxygen to give ^1^O_2_,^1^ a highly reactive oxygen species (ROS).^2^ This type II photodynamic reaction is believed to be the major pathway for photodynamic antimicrobial chemotherapy.^3^ A second pathway, the type I photodynamic reaction, involves the transfer of energy to a substrate such as a cell membrane; from this, the transfer of a hydrogen atom to form radicals takes place. The radicals then react with oxygen to form oxygenated products.^2^ When the energy is transferred the PS is regenerated and acts as a catalyst, meaning that many ^1^O_2_ molecules can be produced as long as a supply of light and molecular oxygen is maintained.^4^

A limited number of investigations have reported the optimization of wavelength for excitation of specific photosensitizers using indirect determinants of ^1^O_2_ production, such as bacterial kill rates,^5^ or tumor size reduction,^6^ and none have reported how the fluence (the rate at which photons irradiate the sensitizer) affects the efficiency of ^1^O_2_ production. Most wavelengths employed in the related field of photodynamic therapy (PDT) are selected due to the tissue penetration depth of the wavelength used,^7^ rather than being necessarily the most efficient wavelength for ^1^O_2_ production. For example, for haematoporphyrin (HpD), the most commonly used photosensitizer for PDT, 630 nm is used for excitation despite this wavelength not being the most efficient for excitation of HpD.^8^ Van Gemert et al. established that the use of green light with HpD may be more efficient at causing tissue necrosis up to a penetration depth of 1.2 mm.^8^

No reports have established the optimization of ^1^O_2_ production from a PS incorporated material by controlling the power of the incident light source. Methylene blue shows an incident light dose‐dependent reduction in both planktonic and biofilm bacteria; however, no correlation with ^1^O_2_ production was reported.^9^ ^1^O_2_ production can be measured using a sacrificial probe such as anthracene‐9,10‐dipropionic acid, disodium salt (ADPA).^10^, ^11^ This probe reacts with ^1^O_2_ to form an endoperoxide, which, unlike ADPA has no strong absorbance at 378 nm. The resulting reduction in absorbance over time can be measured and correlated with the production of ^1^O_2_.^12^ Lindig et al. first reported the use of ADPA as a method of ^1^O_2_ detection in 1980.^13^ Previous to this 1,3‐diphenylisobenzofuran (DPBF) had been used to monitor ^1^O_2_ production. DPBF is water insoluble, so could not be used to measure ^1^O_2_ production in systems where water was a large component. ADPA is water soluble and therefore can be used to measure ^1^O_2_ production in aqueous systems.^13^

**Figure 1:**
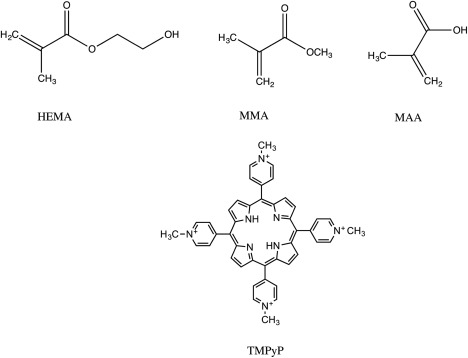
Chemical structures of the monomers and photosensitiser used in this study. A random copolymer of the monomers was produced by free radical polymerization.

Recently, we described the development of a method for quantifying ^1^O_2_ production from a porous, medically relevant hydrogel. A proportion of the probe is absorbed into the hydrogel structure, and this must be accounted for to allow calculation of the rate of ^1^O_2_ production.^14^ The hydrogel is a random copolymer of 2‐hydroxyethyl methacrylate, methyl methacrylate, and methacrylic acid (HEMA:MMA:MAA), onto the surface of which is imbibed the photosensitizer, TMPyp (Figure 1). In this study we exploit this method to address the question of whether application of the same total energy while varying illumination power and time affects the rate of ^1^O_2_ production from a TMPyP‐incorporated HEMA:MAA:MMA polymer. The efficiency of ^1^O_2_ production from two light sources with differing spectral outputs is compared.

## METHODS AND MATERIALS

### Materials

Tetrakis(4‐*N*‐methylpyridyl)porphyrin (TMPyP) was obtained from Tokyo Chemical Industries (Japan). 2‐hydroxyethyl methacrylate ≥99% (HEMA), methyl methacrylate 99% (MMA), methacrylic acid 99% (MAA), ethylene glycol dimethacrylate 98% (EGDMA), and benzoyl peroxide 70% (BPO) were obtained from Sigma‐Aldrich (UK). Anthracene‐9,10‐dipropionic acid, disodium salt >98% (ADPA) was obtained from Chemodex (Switzerland). All materials were used as supplied.

### Hydrogel synthesis

Copolymers consisting of HEMA:MMA:MAA were synthesized as described previously.^15^ Briefly, a 10 g solution of 80 : 10:10 w/w HEMA:MMA:MAA (7.888 g: 0.986 g: 0.986 g), with 0.4% w/w BPO (0.04 g) as the initiator and 1% w/w EGDMA (0.1 g) as the crosslinker were mixed by stirring, then injected into a plate mold. Polymerization proceeded for 2 h in a fan‐assisted oven set at 90**°**C. The resulting film was soaked in deionized water for 14 days to allow leaching of any unreacted monomer. Figure 1 shows the structures of the monomers and photosensitizer used in this study.

### Loading of hydrogel with TMPyP

A solution of 100 μg/mL TMPyP in phosphate buffered saline (PBS) was prepared. Samples, 5 × 20 mm, were dipped in 10 mL TMPyP solution for 2 min to allow electrostatic attachment of the photosensitizer onto the surface of the hydrogel.^15^ The samples were washed and then soaked in deionized water for 7 days.

### Determination of output spectrum and the power of LED array and halogen illumination sources

The power outputs of an adjustable output white LED array (97 W, (Flolight™, Microbeam 1024 daylight spot, Markertek, UK) and two white halogen bulbs (250 W, Radium Ralogen® TD bulbs, Radium Lampenwerk GmbH, Wipperfürth, Germany) were measured using an Ocean Optics Jaz spectrometer (Ocean Optics, Winter Park, FL) and SpectraSuite software. The LED array was held at 11 cm above the spectrometer. The halogen source was positioned to give the same power output between 500 and 600 nm as the LED array. The halogen source was held at 6 cm above the spectrometer to achieve a power of 3.02 mW/cm^2^, 9 cm to achieve 2.11 mW/cm^2^, 13 cm to achieve 1.51 mW/cm^2^, and 27 cm to achieve 0.60 mW/cm^2^. TMPyP has a UV‐visible absorption profile characterised by a Soret band at 430 nm and three Q bands between 500 and 600 nm. The two light sources had differing spectral outputs allowing determination of the most efficient wavelength of excitation for ^1^O_2_ generation.

#### ^1^O_2_ quantification at varying times and illumination powers

ADPA was dissolved in a small amount of methanol and agitated until dissolved. This was added to a solution of methanol:water (20:80 v/v) and optical density at 378 nm was adjusted to 0.3, using the same solvent mixture, using a Cary 50 scan UV‐Visible spectrophotometer. Five separate replicate samples of hydrogel (5 × 20 mm^2^) were placed in a UV cuvette containing 4 mL ADPA solution. Light was applied using either an adjustable power output white LED array held at 11 cm above the samples, or the white halogen source held at varying heights to give equivalent power outputs, with a fan to avoid heating. The total light dose (energy) was kept constant at 50.48 J/cm^2^. The time points required to give the same total energy in each experiment were calculated as follows; 100% power – 140 min, 75% power – 187 min, 50% power – 280 min, 35% power – 400 min, 25% power – 560 min, 10% power – 1400 min. The time points for each illumination power were as follows: 100% power – 0, 20, 40, 60, 80, 120, 140 min; 75% power – 0, 25, 50, 75, 100, 125, 150, 186 min; 50% power – 0, 40, 80, 120, 160, 200, 240, 280 min; 35% power – 0, 60, 120, 180, 240, 300, 360, 400 min; 25% power – 80, 160, 240, 320, 400, 480, 560 min; 10% power – 0, 180, 360, 540, 720, 900, 1080, 1260, 1400 min. The rate of production of ^1^O_2_ was obtained from plots of ADPA absorbance values at 378 nm, in the form of ln (*A*/*A*_0_) against time, where *A* is absorbance at time *t* and *A*_0_ is absorbance at time 0. The rate of ADPA uptake into the porous copolymer, where observed, was characterized from the slope of these plots by subtracting the rate of reduction in absorbance of ADPA in the dark from the rate of reduction in absorbance of ADPA in the light.^14^

## RESULTS

### Power output of visible light sources

To assess the effect of changing the illumination power on ^1^O_2_ generation while maintaining the total energy supplied at 50.48 J/cm^2^, a number of different powers were used. The maximum power supplied by the LED array was 6.04 mW/cm^2^ (integrating between 500 and 600 nm), and is denoted hereafter as 100% power. 75, 50, 35, 25, and 10% powers, corresponding to 4.53, 3.02, 2.11, 1.51, and 0.60 mW/cm^2^ respectively were also used as irradiation conditions. The maximum output from the halogen source was lower, and therefore only 50, 35, 25, and 10% powers, corresponding to 3.02, 2.11, 1.51, and 0.60 mW/cm^2^, were studied using this source. The percentage powers and equivalent powers in mW/cm^2^ are shown in Table 1, and the percentage powers are used to describe the power output from the light sources throughout this study.

**Table 1:** The Percentage of Total Power and the Measured Power Outputs of the White LED Array and the White Halogen Source Between 500 and 600 nm

Percentage power output	Measured power output (mW/cm^2^)
100	6.04
75	4.53
50	3.02
35	2.11
25	1.51
10	0.60

The halogen source was found to have a lower maximum power output between 500 and 600 nm and so would be expected to produce lower ^1^O_2_ yields than that of the LED array.

**Figure 2:**
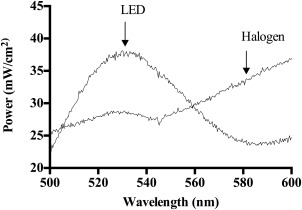
Measured power outputs of a white LED array and a white halogen source, integrated between 500 and 600 nm.

**Figure 3:**
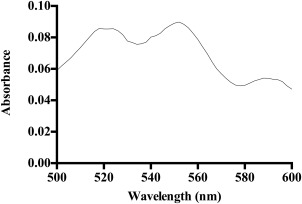
UV‐visible spectrum of the Q bands of TMPyP incorporated in a HEMA:MAA:MMA copolymer between 500 and 600 nm.

The power outputs of the LED array and the halogen source, integrated between 500 and 600 nm (Figure 2) were measured to determine if the power outputs of the two light sources varied along the wavelength range of the Q bands of TMPyP (500–600 nm), which are responsible for the production of ^1^O_2_. Both light sources were set to produce a total power of 3.02 mW/cm^2^ (50% of the LED maximum power) over this wavelength range. The Q bands of TMPyP, which are responsible for the production of ^1^O_2_ lie within this range (500–600 nm), as shown in the UV‐visible spectrum of TMPyP incorporated HEMA:MAA:MMA copolymer, Figure 3.

The LED array produced a higher power at wavelengths corresponding to two of the Q bands of TMPyP, with *λ*_max_ values of 520 nm and 556 nm. The halogen source produced a higher power at the wavelength responsible for the Q band of TMPyP with a *λ*_max_ of 590 nm. If the Q bands at 520 nm or 556 nm are responsible for the majority of ^1^O_2_ production the LED array would be expected to be most efficient for ^1^O_2_ production, if the Q band at 590 nm is the most efficient wavelength for ^1^O_2_ production the halogen source would be expected to be most efficient for ^1^O_2_ production. This is an important finding, as there is no literature reporting how wavelength‐specific excitation of Q bands affects singlet oxygen production rates. Initial excitation by an incident photon populates excited singlet states (*S*_2_ for Soret excitation and *S*_1_ for Q band excitation).^16^ Following population of these states, internal conversion rapidly takes place to the lowest vibrational level of each state.^17^ Singlet oxygen production then depends entirely on the empirical kinetics of intersystem crossing from *S*_1_ to *T*_1_. The differences observed herein therefore suggest that more efficient population of *S*_1_ takes place at 590 nm. According to the Franck Condon principle, internuclear separation between the molecules in the ground vibrational state of *S*_0_ and the ground vibrational state of *S*_1_ is therefore minimal for this transition relative to other *S*_0_ – *S*_1_ transitions from/to other vibrational levels, and therefore the cascade which leads to singlet oxygen production proceeds most efficiently from excitation at this wavelength.

### Quantification of ^1^O_2_ generation at varying times and illumination powers for a white LED array

**Figure 4:**
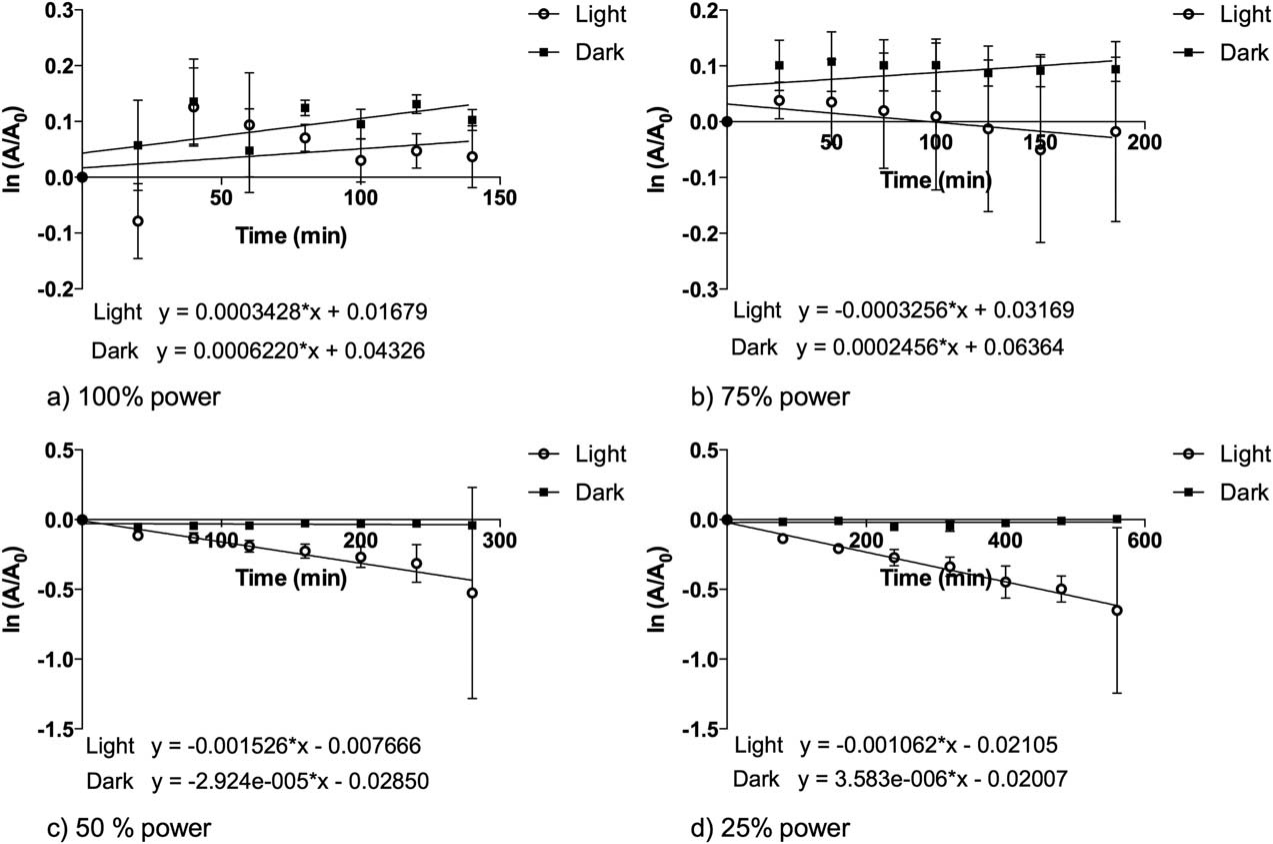
First order plots for reduction in ADPA absorbance by ^1^O_2_ produced from TMPyP incorporated HEMA:MMA:MAA polymer at 378 nm following either irradiance, using an LED array, or dark conditions with varying power and total times. The power outputs and time points used in the experiment were as follows: (a) 100% power – (6.04 mW/cm^2^), 20, 40, 60, 80, 120, 140 min; (b) 75% power – (4.53 mW/cm^2^), 25, 50, 75, 100, 125, 150, 186 min; (c) 50% power – (3.02 mW/cm^2^), 40, 80, 120, 160, 200, 240, 280 min (d) 25% power – (1.51 mW/cm^2^), 80, 160, 240, 320, 400, 480, 560 min, where *A* is the absorbance at time *t* and *A*_0_ is the absorbance at time 0.

The reduction in ADPA absorbance at 378 nm was measured using UV‐visible spectroscopy. Figure 4 shows the reduction in ADPA absorbance at 378 nm as a function of time using the LED array at various power outputs. The reduction in ADPA absorbance is proportional to the production of ^1^O_2_; as ^1^O_2_ is produced it reacts with ADPA to produce an endoperoxide, which has no absorbance at 378 nm due to breakdown of the anthryl chromophore.^18^ The same total energy, 50.48 J/cm^2^ between 500 and 600 nm, was supplied in each experiment to segments by varying the illumination power and time. The reduction in absorbance of ADPA when the maximum power of the LED array was applied, as well as at 75, 50, and 25% of the total power, was determined with time. Controls, where the TMPyP incorporated HEMA:MAA:MMA polymer was placed in a cuvette along with the ADPA:methanol:water solution, and kept in the dark, were also characterized in the same way.

First order plots for 100% and 75% power showed little change in absorbance, showing that little or no ^1^O_2_ was being produced. The first order plot for 25% power showed the largest drop in absorbance at 378 nm, which corresponds to the highest rate of production of ^1^O_2_. Overall, illumination using the LED source led to negligible ^1^O_2_ production from the TMPyP incorporated copolymer.

### Quantification of ^1^O_2_ generation at varying times and illumination powers for a white halogen source

**Figure 5:**
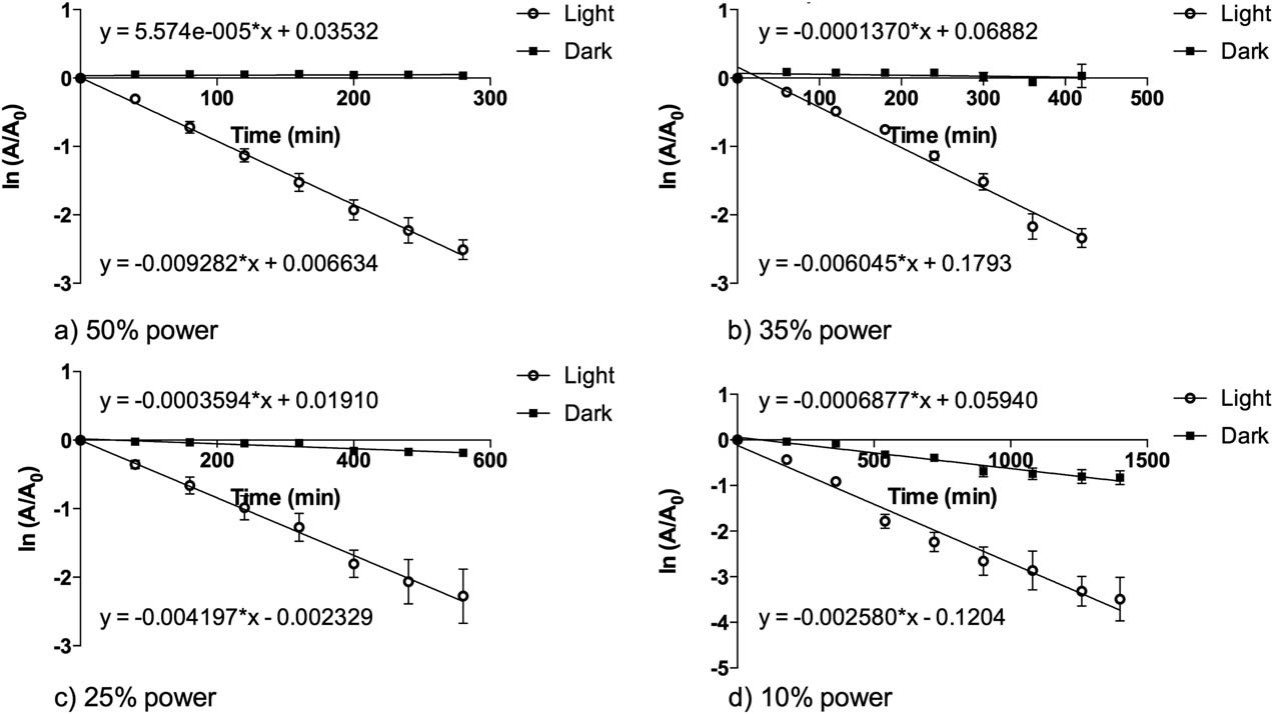
First order plots for reduction in ADPA absorbance by ^1^O_2_ produced from TMPyP‐incorporated HEMA:MMA:MAA polymer at 378 nm following either irradiance using the white halogen source or dark conditions with varying power and total times. The power outputs and time points used in the experiment were as follows: (a) 50% power (3.02 mW/cm^2)^ ‐ 40, 80, 120, 160, 200, 240, 280 min; (b) 35% power (2.114 mW/cm^2^) – 0, 60, 120, 180, 240, 300, 360, 400 min; (c) 25% power (1.51 mW/cm^2^) ‐ 80, 160, 240, 320, 400, 480, 560 min; (d) 10% power (0.604 mW/cm^2^) – 0, 180, 360, 540, 720, 900, 1080, 1260, 1400 min, where *A* is the absorbance at time *t* and *A*_0_ is the absorbance at time 0.

Figure 5 shows the reduction in ADPA absorbance at 378 nm as a function of time using the halogen source at various power outputs. The same total energy, 50.48 J/cm^2^ between 500 and 600 nm, was supplied with irradiance power and time being varied. The power outputs used were calculated based on the LED power output, to allow the two to be comparable. The halogen source did not produce as high a maximum power as the LED and so the highest power used for the halogen source was 50% of the LED maximum power. In order to allow easy comparison between the two light sources the maximum output of the halogen source (3.02 mW/cm^2^) is referred to throughout as 50% power. The power outputs used were as follows, 3.02 mW/cm^2^ (50% of the LED maximum power), 2.11 mW/cm^2^ (35% of the LED maximum power), 1.51 mW/cm^2^ (25% of the LED maximum power), and 0.60 mW/cm^2^ (10% of the LED maximum power).

Varying the power and time to give the same total energy produced similar overall yields of ^1^O_2_ when the white halogen source is used. Figure 5(d) shows a reduction in the dark control samples. It is known that this is due to absorption of ADPA into the hydrogel.^14^ The rate of production of ^1^O_2_ was obtained from plots of ADPA absorbance values at 378 nm, in the form of ln (*A*/*A*_0_) against time, where *A* is absorbance at time *t* and *A*_0_ is absorbance at time 0. Uptake of ADPA in the copolymer, where observed, was accounted for by subtraction of the rate of reduction in absorbance in the dark from the rate of reduction of absorbance in the light, giving a true rate of production of ^1^O_2_.

### Calculation of the rate of ^1^O_2_ production by the white LED array and the white Halogen source at various powers and illumination times

**Figure 6:**
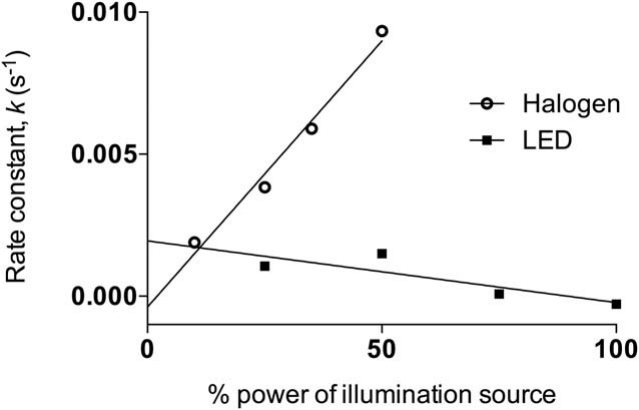
A plot of rate constant of ^1^O_2_ production as a function of power output from TMPyP incorporated HEMA:MMA:MAA copolymer at 378 nm with varying power and total times. The power outputs used in the experiment were as follows: 100% power, 6.04 mW/cm^2^; 75% power, 4.53 mW/cm^2^; 50% power, 3.02 mW/cm^2^; 35% power, 2.114 mW/cm^2^; 25% power, 1.51 mW/cm^2^; 10% power, 0.604 mW/cm^2^. The rate of reduction of absorbance for the 10% power using the halogen source has been compensated for by subtraction of the dark rate from the light. The power sources used were a white LED array or a white halogen source.

The gradient of the first order plots of the white halogen source can be used to obtain the rate constants, *k* for the production of ^1^O_2_. Figure 6 shows the rate constants of the varying power outputs and their linear correlation with the irradiance powers.

A linear relationship between increasing power and singlet oxygen production was observed when a TMPyP incorporated HEMA:MAA:MMA hydrogel was illuminated with the white halogen source. This relationship is important to quantify, as it indicates, within the power range studied, that overall quantum yield for ^1^O_2_ production does not vary. The relationship between power and ^1^O_2_ production for this TMPyP‐incorporated hydrogel can be used as an indicator of the likely relationship between increasing power and ^1^O_2_ production for any PS‐incorporated hydrogel. A negligible amount of ^1^O_2_ is produced when the same material is illuminated with the white LED array.

## DISCUSSION

The rate of reduction of absorbance of ADPA solution is directly proportional to the quantum yield of ^1^ $O_{2.}^{10,}$ 11 As ^1^O_2_ is produced it reacts with the ADPA to produce an endoperoxide, less ADPA present will give a lower absorbance reading.^19^ As illustrated in Figure 2 the LED array produces a higher power output at the Q bands for TMPyP at 520 nm and 556 nm. It has been reported that irradiation at 430 nm (TMPyP Soret band) and between 500 and 600 nm (Q bands of TMPyP) can produce ^1^O_2_ in TMPyP.^20^ If the Q bands for TMPyP at 520 nm and 556 nm were responsible for the highest quantum yield of ^1^O_2_ it would be expected that the LED array would show a high ^1^O_2_ generation rate constant and a large reduction in the absorption of ADPA. This is not observed and therefore, it can be concluded that the Q bands at 520 nm and 556 nm are not the most efficient generators of ^1^O_2_ in TMPyP. The white halogen source produces a much higher yield of ^1^O_2_ at every power than that produced by the LED array. The overall yield of ^1^O_2_ produced by illumination with the halogen source for each power output is similar.

The power output of the LED array and the halogen source was measured between 580 and 600 nm, the range corresponding to the longest wavelength Q band of TMPyP.

The halogen source provided 0.852 mW/cm^2^ power between 580 and 600 nm, which equates to 1.8 times the irradiance power of the LED array (0.48 mW/cm^2^). This wavelength corresponds to the longest wavelength Q band of TMPyP and provides further evidence that the Q band at 590 nm for TMPyP is the most efficient for ^1^O_2_ production. Knowledge of the most efficient wavelength and light source to select, to provide the most efficient production of ^1^O_2_ will allow more efficient photosensitisation in, for example, photodynamic therapy and photodynamic materials.^21^

In the power range studied, the rate of ^1^O_2_ production is linearly related to the power applied. The halogen source is more efficient at producing ^1^O_2_ than the white LED array. The highest rate of ^1^O_2_ production is achieved when an illumination power of 3.02 mW/cm^2^, integrated between 500 and 600 nm is applied for 280 min, using the white halogen source. An understanding of the effect power and time have on the rate of ^1^O_2_ production is useful to allow selection of the optimal wavelength and irradiation power for a photosensitizing hydrogel. Consideration of the intended use of the material is important. For example, with optical applications, such as an intra‐ocular lens for cataract replacement surgery, a high power would not be usable as damage to the eye may occur. It is useful for a clinician to be able to quantify what power and time will give the same yield of ^1^O_2_ but at a lower, less potentially damaging power.

## CONCLUSIONS

The Q band of TMPyP at 590 nm, while not the only wavelength ^1^O_2_ is produced at, is the most efficient for production of ^1^O_2_. For production of ^1^O_2_ from a TMPyP incorporated HEMA:MAA:MMA polymer, the white halogen source is more suitable than the white LED array. The highest rate of production is achieved when the polymer is irradiated with a fluence rate of 3.02 mW/cm^2^ for 280 min. This study shows a linear relationship between increasing illumination power and production of ^1^O_2_, when an appropriate illumination source is chosen. The linear relationship found in this study can be applied to predict the likely effect increasing illumination power will have on any PS‐incorporated hydrogel. Selection of an illumination source with a high power output over the wavelength range responsible for the most efficient production of ^1^O_2_ will increase the photosensitization effect of the PS‐incorporated material.
